# A randomized controlled study on an integrated approach to prevent and treat re-adhesion after transcervical resection of moderate-to-severe intrauterine adhesions

**DOI:** 10.6061/clinics/2021/e1987

**Published:** 2021-04-26

**Authors:** Li-Zhen Pan, Ying Wang, Xian Chen

**Affiliations:** Department of Obstetrics and Gynecology, Nanping People's Hospital to Fujian University of Traditional Chinese Medicine, Nanping 353000, Fujian, China

**Keywords:** Moderate-to-severe Intrauterine Adhesions, Transcervical Resection of Adhesion, Intrauterine Perfusion, Danshen injection, Re-adhesion

## Abstract

**OBJECTIVES:**

This study aims to compare the clinical efficacy of an integrated approach to prevent and treat the recurrence of moderate-to-severe intrauterine adhesions (IUA) after hysteroscopic transcervical resection of adhesion (TCRA).

**METHODS:**

The study included a total of 70 patients with moderate-to-severe IUAs who underwent TCRA. Patients were randomly divided into two groups: treatment group (n=35) and control group n=35). In the treatment group, patients underwent balloon uterine stent placement and artificial cycle as well as received intrauterine perfusion of Danshen injection and oral Chinese medicine. In the control group, patients underwent balloon uterine stent placement and artificial cycle as well as received hyaluronic acid sodium and intrauterine device (IUD). Follow-up was performed after treatment of uterine cavity, menstruation and pregnancy.

**RESULTS:**

After 3 months of treatment, we observed a significantly lower rate of intrauterine re-adhesion (45.71% *versus* 77.14%, *p*=0.044) and significantly higher clinical efficiency (82.86% *versus* 77.14%, *p*=0.025) in the treatment group than those in the control group. After 6 months of treatment, we observed a significantly higher clinical efficiency in the treatment group than that in the control group (88.57% *versus* 68.57%, *p*=0.039). During the follow-up period, the pregnancy rate was 45.71% and 37.14% in the treatment group and control group, respectively, although the difference was not statistically significant (*p*=0.628).

**CONCLUSIONS:**

After surgical management of IUA, the integrated treatment combining a uterus stent placement and artificial cycle with Danshen injection and oral Chinese medicine can improve the condition of menstruation, and prevent and treat recurrence of IUA.

## INTRODUCTION

Intrauterine adhesion (IUA) is characterized by reduced menstrual blood volume, amenorrhea, infertility, and recurrent abortion (1), which seriously affects the reproductive health of women. Hysteroscopy is the preferred diagnostic method for IUAs; transcervical resection of adhesion (TCRA) is the standard surgical procedure to treat IUAs and plays an irreplaceable role in separating IUAs (2). However, IUAs are prone to recurrence after surgery. There is no effective treatment to prevent and treat recurrent IUAs so far. The current expert consensus on the diagnosis and management of IUAs (3) recommends the post-TCRA use of intrauterine device (IUD), intrauterine support balloon, and bioadhesive material to prevent re-adhesion, and estrogen, amniotic membrane (4), stem cell (5), and other synthetic therapies to promote endometrial regeneration and repair. However, there is no effective prevention and control method for severe IUA. The present study aims to explore the clinical efficacy of the integrated therapy combining Chinese oral medicine and intrauterine perfusion of Danshen injection for the prevention and treatment of recurrent IUA after TCRA for moderate-to-severe IUAs.

## MATERIALS AND METHODS

### Subjects

The present study included a total of 70 patients with moderate-to-severe IUAs who underwent TCRA in our hospital from January 1, 2015 to December 31. Patients were divided into two equal groups selected using random number table: treatment group (n=35) and control group (n=35). The average age of patients in the treatment group was 33.06±5.07 years. There were 23 patients with moderate IUA and 12 patients with severe IUA. Two patients with severe IUA had a chief complaint of amenorrhea. Furthermore, 31 patients had significantly reduced menstrual blood volume, and six patients had secondary infertility. The average age of patients in the control group was 32±6.71 years. There were 22 patients with moderate IUA and 13 patients with severe IUA. One patient with severe IUA had a chief complaint of amenorrhea. Furthermore, 30 patients had significantly reduced menstrual blood volume, and seven patients had secondary infertility. There were no statistically significant differences in general data between the groups (*p*>0.05, Table 1). This study was conducted in accordance with the declaration of Helsinki and was approved by the Ethics Committee of Nanping People’s Hospital. Written informed consent was obtained from all participants.

### Hysteroscopic diagnostic criteria

Hysteroscopy was used for the diagnosis of IUAs. The following diagnostic criteria were used for mild IUA (6): i) adhesions affecting less than one-fourth of the uterine cavity, ii) thin or fine adhesions, and iii) very mild or clearly visible lesions in the oviduct opening and in the upper part of the uterine cavity. The following diagnostic criteria were used for moderate IUA (6): i) adhesions affecting one-fourth to three-fourth of the uterine cavity, ii) merely solitary adhesions, iii) no adhesion of the uterine wall, and iv) closure of the oviduct opening and part of the upper end of the uterine cavity. The following diagnostic criteria were used for severe IUA (6): i) adhesions affecting more than three-fourth of the uterine cavity, ii) adhesions of the uterine walls, iii) thick adhesions, and iv) complete closure of the oviduct opening and upper end of the uterine cavity.

### Inclusion criteria

The inclusion criteria were as follows: i) patients satisfying the hysteroscopic diagnostic criteria for moderate-to-severe IUA; ii) patients diagnosed with kidney deficiency and blood stasis according to the syndrome differentiation in traditional Chinese medicine (TCM) (7); iii) patients with any two of the following primary manifestations: significant reduction in recent menstrual blood volume, exhaustion after a light menstrual bleeding, amenorrhoea, dark purple colored menstrual blood, menstrual clots, and less than 2 days of menstruation; iv) patients with the any two of the following secondary symptoms: abdominal pain during menstruation, weakness at the waist and knees, insomnia, forgetfulness, dizziness and tinnitus, occasional premenstrual mammary swelling, mental fatigue, and lack of strength; v) patients with a pale face; vi) patients with the following tongue and pulse signs: the tongue was dim or light red, or presented with ecchymosis or petechia, and the pulse was wiry-thready or deep; vii) patients aged between 20-40 years; viii) patients with reproductive demand; ix) patients with normal levels of the six sex hormones; and x) patients who agreed with the treatment and provided a signed informed consent.

### Exclusion criteria

The exclusion criteria were as follows: i) patients allergic to Salvia miltiorrhiza (Danshen) injection, ii) patients with inflammation of the genital tract, iii) patients with pelvic inflammation, iv) patients with malignant tumors of reproductive systems, v) patients with endometrial tuberculosis, vi) patients with uterine malformations, and vii) patients who took sex hormones in the past 3 months.

### Surgical methods

The time of operation was set at 3-7 days after the end of menstruation. In patients with amenorrhea, 400 g of vaginal misoprostol was administered 8-10h preoperatively. A Sopro-Comeg parallel vision hysteroscopy endo-operative system HEOS (Sopro-Comeg, La Ciotat, France) was used to detect IUAs. The mechanical separation of IUA was performed under general anesthesia with endotracheal intubation or intravenous anesthesia. The normal shape of the uterine cavity was restored as far as possible, and the blockage of the bilateral oviduct openings were removed. Thirteen patients with infertility underwent a laparoscopic surgery. Eight patients underwent an operation guided by ultrasound. Forty-nine patients underwent a hysteroscopic surgery.

### Treatment

Postoperatively, patients in the treatment group were slowly injected with 5 mL Danshen injection (Harbin Pharm Group Sanjing Pharmaceutical Co, Ltd., Heilongjiang, China) into the uterine cavity through a disposable tube. After 15 min, the tube was removed, and a balloon uterine stent (Cook Inc., Bloomington, Indiana, USA) was placed; then, 5 mL normal saline was injected into the balloon. Patients took 20 mg metronidazole and vitamin B6 tablets orally *bid* and underwent vaginal scrubbing with a small amount of iodophor *qd*. On the fifth day after removing the balloon stent, if the vaginal bleeding stopped, the first cycle of intrauterine perfusion of Danshen injection was started. Intrauterine perfusion was performed once every 3 days for a total of three times per cycle. The second cycle began 3 days after the patient’s menstrual bleeding stopped completely, and patients underwent three cycles of intrauterine perfusion in total. Before performing intrauterine perfusion, it was confirmed that the patient did not have genital tract inflammation and bleeding. Postoperatively, patients in the control group were slowly injected with 2 mL sodium hyaluronate gelatin in the uterine cavity. Then, a balloon uterine stent was placed (Cook Inc., Bloomington, Indiana, USA). After 5 days, the balloon stent was removed, and a GT300 IUD (Chongqing Medical Equipment Factory, Chongqing, China) was placed. From postoperative day 1 onwards, patients in both groups received estradiol valerate (Progynova, 3 mg/day) orally for 21 days. Additionally, they took dydrogesterone (Duphaston, 10 mg/day) in the last 10 days. Then, the medication was discontinued until the onset of menstruation. The next cycle of the medication began on the fifth day of menstruation, and patients received medication for a total of three cycles. Additionally, patients in the treatment group took a kidney-tonifying, blood-nourishing, and endometrium-regulating decoction. The decoction was prepared by boiling the following herbs in water: 20 g of Cuscuta, 15 g of raspberry, 15 g of Herba Cistanches, 20 g of mulberry fruit, 20 g of barbary wolfberry fruit, 20 g of prepared *Rehmannia* root, 15 g of donkey-hide gelatin, 10 g of Chinese angelica, 10 g of *Atractylodes macrocephala*, 10 g of tree peony bark, and 6 g of *Achyranthes bidentata*. Patients took a single dose of the decoction twice every day. The administration of the decoction began on the third day after patient’s menstrual bleeding stopped completely and continued further to complete a course of 10 days; patients took the decoction for a total of three courses.

### Follow-up

The occurrence of adverse reactions during the treatment was recorded in both the groups. After continuing the treatment for three menstrual cycles, menstruation parameters were evaluated. Hysteroscopy was performed to assess the recovery of the uterine cavity 3-7 days after patient’s menstrual bleeding stopped completely. Then, IUDs were removed in the control group, and menstruation and pregnancy in both groups were followed-up for 6 months after the treatment.

### Evaluation of clinical curative effect

A patient was considered cured if her menstruation including menstrual blood volume returned to normal after the treatment. A patient was considered improved if her menstruation returned to normal with a reduced menstrual blood volume after the treatment. The treatment was considered ineffective for the patient if there was no significant improvement in menstruation after the treatment compared with that before the operation. The total effective rate was calculated as the sum of the cure rate and the improvement rate.

Clinical curative effect of the treatment was evaluated as recovered, effective, or invalid based on previously reported criteria (8,9): Recovered: the menstruation gradually recovered from beubf absent to present, and from small volume to normal volume; the uterine cavity was normal under a hysteroscope, the surface of the endometrium was smooth, and no adhesions were found; the bilateral cornua uteri and oviduct openings were clearly visible. Effective: the menstrual volume increased, but the volume remained less than the normal level; the uterine cavity was basically normal under a hysteroscope, and the degree and scope of adhesions were significantly reduced when compared with that before the operation, but there were still adhesions in part of the uterus. Ineffective: The menstruation was not restored, the menstrual volume did not improve, and recurrent adhesions could be observed under the hysteroscope, which did not significantly change, when compared with those before the operation. The total effective rate was calculated as the sum of the recovered rate and effective rate.

### Statistical analysis

All statistical analyses were performed using SPSS 21.0 software (IBM, Armonk, NY, USA). Continous variables data are expressed as mean±standard deviation, whereas categorical variables are expressed as percentage (%). Data were compared between the groups using the chi-square test. Ranked data were evaluated using a rank sum test. We calculated *p*-values using a two-tailed test. A *p*-value of <0.05 was considered statistically significant.

## RESULTS

### Intrauterine re-adhesion at 3 months after the treatment

A total of 70 patients participated in the study. After 3 months of treatment, the treatment group showed a significantly lower intrauterine re-adhesion rate than the control group (45.71% (16/35) *versus* 77.14% (27/35), *X* ^2^=8.106, *p*=0.044, Table 2).

### Clinical curative effect at 3 months after the treatment

The treatment group showed significantly higher total effective rate than the control group (82.86% (29/35) *versus* 77.14% (27/35), *p*=0.025, Table 3).

### Menstruation and pregnancy at 6 months after the treatment

At 6 months after the treatment, the treatment group showed significantly higher total menstrual recovery rate than the control group (88.57% (31/35) *versus* 68.57% (24/35), *p*=0.039, Table 4). In the treatment group, the pregnancy rate was 45.71% (16/35), and two patients had an abortion during early pregnancy. In the control group, the pregnancy rate was 37.14% (13/35), and one patient had an abortion during early pregnancy. The difference in pregnancy rate was not statistically significant between the two groups (*p*=0.628, Table 4).

### Adverse reactions during the treatment

In both the groups, no balloon uterine stent fell off, and no patient developed drug allergy. However, only one patient in the treatment group developed fever. Furthermore, vaginal infection was found in two patients in the treatment group and one patient in the control group.

## DISCUSSION

### Perplexity for the treatment of IUAs

Pathogenesis of IUA remains unknown although there are several hypotheses on this, including active proliferation of fiber cells (10) and neural reflection (3). The risks of IUAs are abortions, pelvic inflammatory disease, IUD related intrauterine diseases, other intrauterine diseases and genetic inheritance (11 -14). Hysteroscopic surgery is the gold standard for the diagnosis and treatment of IUA. According to a previous report, the recurrence rate of IUA and severe IUA after TCRA is 3.1-23.5%, and 20.0-62.5%, respectively (15). The prevention of postoperative re-adhesion and the promotion of endometrial repair are key to treating IUAs. The comprehensive therapy combining intrauterine Foley balloon placement, IUD placement, and estrogen administration is classically used to prevent and treat postoperative re-adhesion (16). The current consensus on the diagnosis and management of IUA points out that (3) the placement of balloon uterine stent in combination with IUD may prevent the postoperative recurrence of IUA by preventing the mutual attachment formation between the wound surfaces *via* the barrier effect. However, it is difficult to completely seal the wound surfaces due to the possible risks of endometrial ischemic necrosis, abnormal bleeding, infection, contraceptive ring incarceration, and uterine perforation. The use of estrogen is the main method to promote endometrial regeneration and repair, but the efficacy of this method depends on whether there is enough residual endometrial tissues and on the dosage of estrogen used (17,18). Many scholars have committed to seek effective methods for preventing and treating recurrence of IUA postoperatively. A previous study reported that intrauterine injection, along with gel barrier, was a relatively effective treatment (19). However, most researchers consider that there is no effective treatment for preventing re-adhesion so far (20,21).

### Feasibility of the intrauterine perfusion of Danshen injection

Bioadhesive materials, such as sodium hyaluronate and polylactic acid gel, have also been used to prevent and treat postoperative recurrence of IUA. Previous studies reported that bioadhesive materials could prevent the formation of IUA *via* the barrier effect, repair the damaged endometrium, inhibit tissue fibrosis, bacteriostasis, hemostasis, and antioxidation, and improve pregnancy rate to a certain extent (22-26). Danshen injection, a Chinese herbal medicine, removes blood stasis and promotes tissue regeneration, and it has been widely used in various branches of medicine. A relatively high intrauterine concentration of Danshen is maintained by intrauterine perfusion in order to improve local blood circulation, destroy bacteria, diminish inflammation, and to promote the loosening and absorption of adhesions. The blunt dissection with bolus injection pressure has become an effective method to prevent and treat postoperative recurrence of IUA (7). A previous study reported that (27) intrauterine perfusion with Danshen injection after the operation of moderate-to-severe IUAs may prevent the recurrence of IUA by inhibiting fibrin aggregation within the endometrium, promoting the dissolution and absorption of fibrin clot, reducing formation of the extracellular matrix, improving local tissue ischemia, and promoting embryo implantation. Danshen injection has many advantages, such as wide range of sources, low price, low toxicity, and few side effects, over bioadhesive materials.

### Analysis of results

In the treatment group, patients were treated with the comprehensive therapy on the 10^th^, 13^rd^, and 16^th^ day of their menstrual cycle, *i.e.*, at the endometrium hyperplasia stage and early secretory stage. Patients underwent intrauterine perfusion of Danshen injection and took kidney-tonifying, blood-nourishing, and endometrium-regulating decoction orally in order to improve the local blood circulation in the uterine cavity, nourish endometrial tissue, and to promote the regeneration and repair of the damaged endometrium. Furthermore, the intermittent intrauterine perfusion may bluntly separate the remaining tiny adhesions, facilitating the attachment of the medicine closely to the wound surface in the uterine cavity, improving the bioavailability of drugs, and achieving the purpose of preventing and treating re-adhesion, reconstructing the endometrium, and restoring menstrual and reproductive functions. After 3 months of treatment, the intrauterine re-adhesion rate and total clinical efficiency in the treatment group were 45.71% and 82.86%, respectively. After 6 months of follow-up, the total menstrual recovery rate in the treatment group was significantly higher than that in the control group (88.57% *versus* 68.57%), whereas both the groups showed similar pregnancy rate. Many previous studies have revealed that (28,29) kidney-tonifying and blood-activating Chinese herbal medicine can improve endometrial blood circulation and the situation of menstruation after for the surgical management of IUA. According to Wang et al. (30), kidney-tonifying and stasis-removing Chinese herbal medicine can promote the proliferation of endometrial stromal cells and reduce their apoptosis by increasing their matrix metalloproteinase 9 (MMP-9) expression, which in turn inhibit the formation of IUA. In the present study, no serious adverse reactions occurred.

In order to minimize the impact of selection bias on the final data, the randomized allocation method and strictly restricted treatment methods were used in the present study. However, the present study still has some limitations. First, our study had a small sample size. Second, the follow-up duration in the present study was short, and the assessment of pregnancy outcomes and adverse reactions requires long-term observation. Third, the findings of this study is not fully explained. We plan to study the mechanisms of treatment efficacy in the future.

## CONCLUSION

The present study compared the clinical efficacy of the integrated approach to prevent and treat re-adhesions of moderate-to-severe IUA after TCRA. Our data suggested that normal menstruation was restored in patients who underwent a uterus stent placement and artificial cycle as well as received Danshen injection and oral Chinese medicine postoperatively. This indicates that the integrated treatment may prevent and treat re-adhesion after TCRA.

## AUTHOR CONTRIBUTIONS

Pan LZ drafted and critically revised the manuscript for important intellectual content. Wang Y and Chen X acquired, analyzed, and interpreted the data. All of the authors approved the final version of the manuscript for publication.

## Figures and Tables

**Table 1:** Baseline characteristics of the two groups of samples.

Groups	Age (years)	Moderate adhesion (n)	Severe adhesion (n)	Reduced menstrual blood volume (n)	Amenorrhea (n)	Infertility (n)
Treatment group (n=35)	33.06±5.07	23	12	31	2	6
Control group (n=35)	32.00±6.71	22	13	30	1	7

**Table 2:** Hysteroscopic evaluation of the intrauterine cavity.

Groups	Normaln (%)	Mildn (%)	Moderaten (%)	Severen (%)	Re-adhesion rate(%)
Treatment group (n=35)	19 (54.29)	6 (17.14)	5 (14.29)	5 (14.29)	45.71
Control group (n=35)	8 (22.86)	14 (40.00)	7 (20.00)	6 (17.14)	77.14

**Table 3:** Clinical curative effect at 3 months after treatment.

Groups	Recovered n (%)	Effective n (%)	Ineffect ive n (%)
Treatment group (n=35)	18 (51.43)	11 (31.43)	6 (17.14)
Control group (n=35)	7 (20.00)	20 (57.14)	8 (22.86)

**Table 4:** Menstruation and pregnancy at 6 months after the treatment.

Groups	Recovered n (%)	Effective n (%)	ineffe ctive n (%)	Pregnancy n (%)
Treatment group (n=35)	15 (42.86)	16 (45.71)	4 (11.43)	16 (45.71)
Control group (n=35)	9 (25.71)	15 (42.86)	11 (31.43)	13 (37.14)
